# Supplementary material for: Association of triglyceride-glucose index with postoperative recovery-related factors in type II cesarean scar pregnancy treated by transvaginal surgery
Source: PeerJ. 2025 Jun 20;13:e19632. doi: 10.7717/peerj.19632 (PMC12184668; doi:10.7717/peerj.19632)
Supplement: Supplemental Information 2 [file peerj-13-19632-s002.docx]

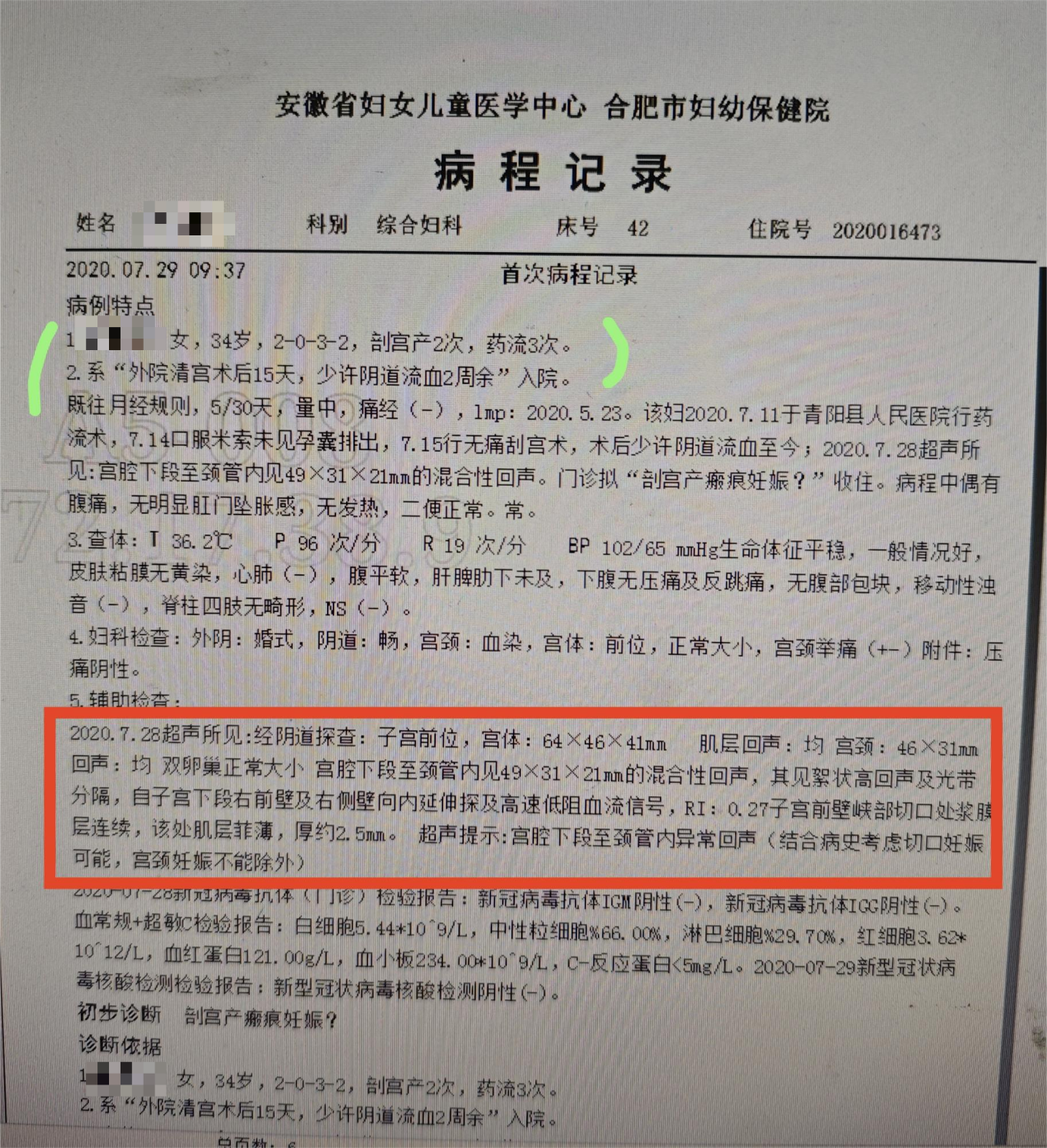


Case Characteristics

Initial Medical Record

1. Female, 34 years old, gravida 2, para 0, abortion 3 times, cesarean section 2 times.

2. Admitted with "light vaginal bleeding for more than two weeks after uterine curettage performed at another hospital 15 days ago."

Past History:

The patient’s menstrual cycle was regular, lasting 5 days every 30 days, with moderate flow and no dysmenorrhea. Last menstrual period (LMP): May 23, 2020. On July 11, 2020, the patient underwent medical abortion at Qingyang County People's Hospital. On July 14, oral misoprostol was administered, but the gestational sac was not expelled. On July 15, painless uterine curettage was performed. Postoperatively, the patient experienced persistent light vaginal bleeding. On July 28, 2020, ultrasound revealed a mixed echogenic mass measuring 49×31×21 mm in the lower uterine segment extending into the cervical canal. She was admitted with a suspected diagnosis of "cesarean scar pregnancy." During the course of the illness, the patient occasionally experienced abdominal pain, without significant rectal pressure sensation, fever, or abnormal bowel and urinary habits.

Vital Signs:

BP: 102/65 mmHg, T: 36.2°C, P: 96 bpm, R: 19 breaths/min. General condition: Stable. Skin and mucous membranes showed no jaundice. Cardiopulmonary examination: Normal. Abdomen: Soft, no tenderness or rebound tenderness, no palpable mass, no shifting dullness. Liver and spleen were not palpable below the costal margin. Spine and limbs: No deformities. Nervous system: Negative.

Gynecological Examination:

- External Genitalia: Normal for marital status.

- Vagina: Patent.

- Cervix: Blood-stained.

- Uterus: Anterior position, normal size, cervical motion tenderness (+/-).

- Adnexa: No tenderness.

Supplementary Examinations:

1. Ultrasound (July 28, 2020):

Transvaginal examination revealed an anteriorly positioned uterus measuring 64×46×41 mm with homogeneous myometrial echogenicity. The cervix measured 46×31 mm with homogeneous echogenicity. Both ovaries were of normal size. A mixed echogenic mass measuring 49×31×21 mm was observed in the lower uterine segment extending into the cervical canal, with flocculent hyperechoic areas and bright bands. High-velocity, low-resistance blood flow was detected extending from the right anterior wall and lateral wall of the lower uterine segment. Resistance Index (RI): 0.27. The serosal layer at the isthmus of the anterior uterine wall was intact, with a thin myometrial layer measuring approximately 2.5 mm in thickness. Ultrasound impression: Abnormal echogenicity in the lower uterine segment extending into the cervical canal (cesarean scar pregnancy is suspected based on the history; cervical pregnancy cannot be excluded).

2. COVID-19 Antibody Test (July 28, 2020):

- IgM: Negative.

- IgG: Negative.

3. Complete Blood Count and C-Reactive Protein (July 28, 2020):

- White blood cells: 5.44×10^9/L

- Neutrophils: 66.00%

- Lymphocytes: 29.70%

- Red blood cells: 3.62×10^12/L

- Hemoglobin: 121.00 g/L

- Platelets: 234.00×10^9/L

- C-reactive protein (CRP): <5 mg/L

4. COVID-19 Nucleic Acid Test (July 29, 2020):

- Negative.

Preliminary Diagnosis:

Cesarean scar pregnancy (CSP)?
